# Long-term feeding issue and its impact on the daily life of congenital diaphragmatic hernia survivors: results of the first patient-led survey

**DOI:** 10.1007/s00383-019-04570-6

**Published:** 2019-11-06

**Authors:** Beverley Power, Soichi Shibuya, Brenda Lane, Simon Eaton, Paolo De Coppi

**Affiliations:** 1 CDH UK, Norfolk, UK; 2 Great Ormond Street Institute of Child Health, University College London, 30 Guilford Street, London, WC1N 1EH UK

**Keywords:** Congenital diaphragmatic hernia, Quality of life, Gastroesophageal reflux, Growth retardation, Multidisciplinary team approach

## Abstract

**Background:**

CDH UK is a registered charity governed by a volunteer committee and providing informal support to patients, families and healthcare workers affected directly or indirectly with congenital diaphragmatic hernia (CDH) internationally. This is the first patient-led survey undertaken by CDH UK aiming for highlighting the feeding problems and their impact on the daily life of CDH survivors.

**Methods:**

Answers from CDH survivors were collected through an online questionnaire (SurveyMonkey^®^) undertaken by CDH UK. The questionnaire contained questions about their feeding problems and support they were receiving for it.

**Main results:**

Overall, 151 patients answered some parts of the survey and 102 patients completed the questionnaire. Overall, 116 (76.8%) responders reported suffering from any type of feeding issue. Gastric acid reflux (GER) and growth retardation were the commonest symptoms experienced by 97 (91.5%) and 72 (62.2%) responders, respectively. Only 18 (17.0%) responders have received any written information on feeding or details of patient/parent support. Eighty (75.5%) responders are satisfied with the level of support they are receiving, but 78 (76.4%) answered that the whole experience associated with the disease has been very or extremely stressful.

**Conclusions:**

CDH survivors frequently have various issues with feeding, which may not be adequately supported or discussed clinically. It is desirable to assist the patients to reliable resources of long-term support, including multidisciplinary team (MDT) approach.

## Introduction

Congenital diaphragmatic hernia (CDH) is one of the most severe congenital malformations managed by pediatric surgeons. Despite significant efforts which have been made to overcome this life-threatening condition, mortality rate remains high at 30% [1–3]. Moreover, patients who survive the disease have high morbidity of postoperative sequala even after discharge. Previous studies identified problems in patients surviving CDH, such as gastroesophageal reflux (GER) and recurrent chest infections, but the impact of these issues on the quality of life (QOL) of patients and their families is unclear and likely to be underestimated.

CDH UK (https://cdhuk.org.uk/) is an international registered charity based in the United Kingdom and one of the largest associations of families affected by a congenital malformation. It is governed by a volunteer committee and has aimed at providing informal support to patients and families facing CDH and their healthcare workers by sharing the experience of other patients and families. Further aim of CDH UK is to contribute to the improvement of the medical treatment by supporting future research and offering information to other organizations on CDH around the world. This patient-led survey was carried out by CDH UK for the purpose of describing the feeding problems of CDH survivors and the impact of them on their QOL.

## Materials and methods

An online questionnaire was organized by CDH UK and sent to registered CDH survivors by email and closed patient forums. The questionnaire contained questions focusing on their feeding problems and support they were receiving (Table 1). Data were collected through an online questionnaire platform (SurveyMonkey^®^) and analyzed by members of CDH UK (BP) in collaboration with pediatric surgeons (PDC, SE, and SS).

**Table 1:** The list of the questionnaire

Q1. Did you/your child receive ECMO support?
Q2. How long were you/your baby in hospital following birth?
Q3. Do you/have you or your child suffered from any type of feeding issue?
Q4. Which type of feeding issue have you or your child suffered?
Q5. How would you describe your/your child’s feeding problems?
Q6. Do you/your child have weight or growth issues?
Q7. Have you/your child been diagnosed with feeding issues?
Q8. Do you agree or disagree with the statement: the feeding issues improved when starting nursery or school?
Q9. Which enteral/parenteral feeding methods are/have been applied to you/your child?
Q10. What is/was the hardest thing to cope with in relation to feeding issues?
Q11. Did you receive any written information on feeding issues or details of patient/parent support?
Q12. Would it have been helpful to have known about the potential for feeding issues antenatally?
Q13. Have you ever received any counselling relating to the feeding issues?
Q14. Have you/your child attended any special clinics?
Q15. Are you satisfied with the level of support that you/your child receives or received relating to the feeding issues?
Q16. How has the feeding issue impacted on your daily life?
Q17. How stressful has the whole experience been?

*ECMO* extracorporeal membrane oxygenation

## Results

### Subjects

**Fig. 1:**
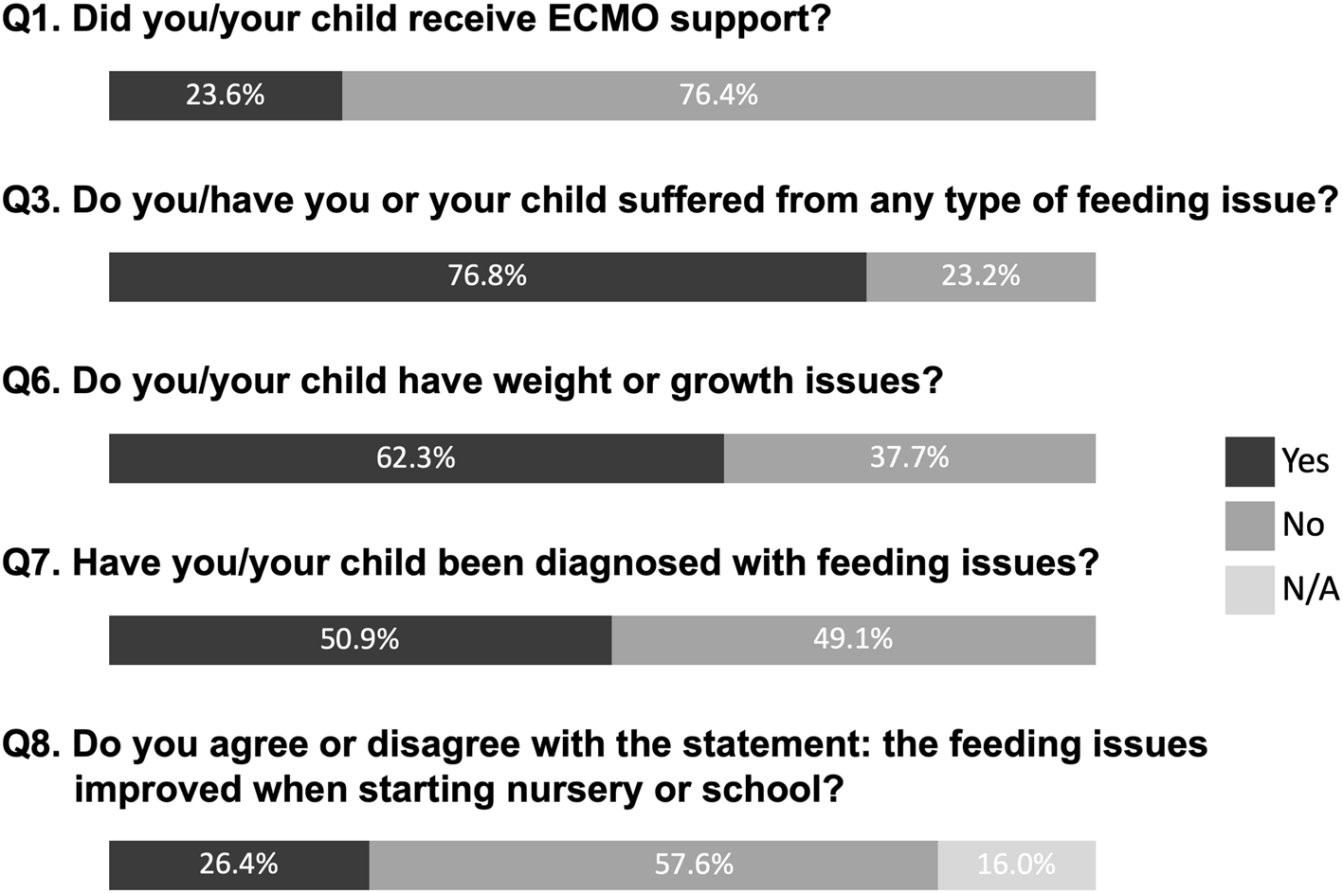
Background information

Overall, 151 families with a patient affected by CDH participated and answered parts of the survey with 106 (67.5%) families completing the questionnaire. Among the participants, 25 (23.6%) patients received extracorporeal membrane oxygenation (ECMO) during resuscitation (Fig. 1; Q1) and 37 (34.9%) patients required hospitalization over 12 weeks after birth.

### Feeding problems

**Fig. 2:**
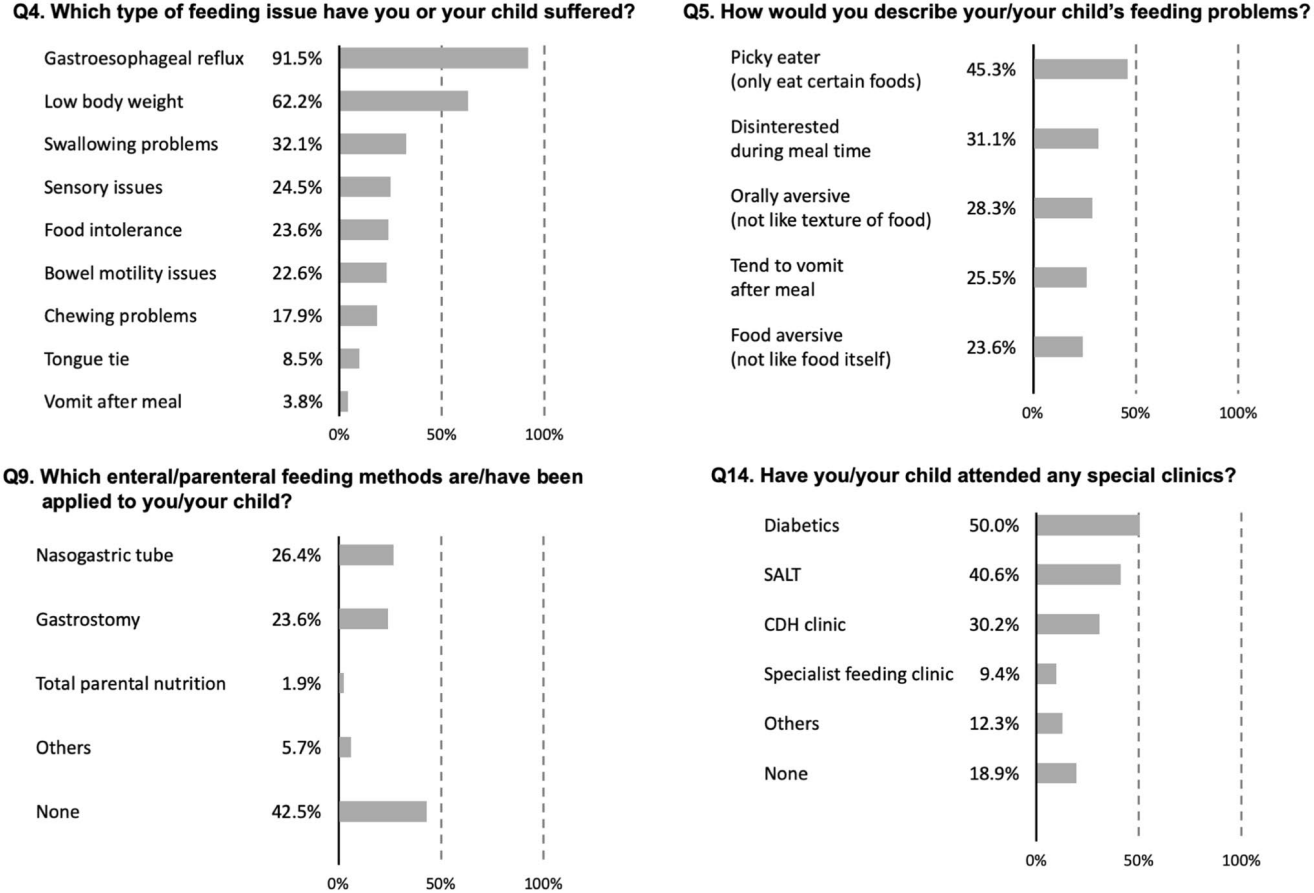
Feeding problems

**Fig. 3:**
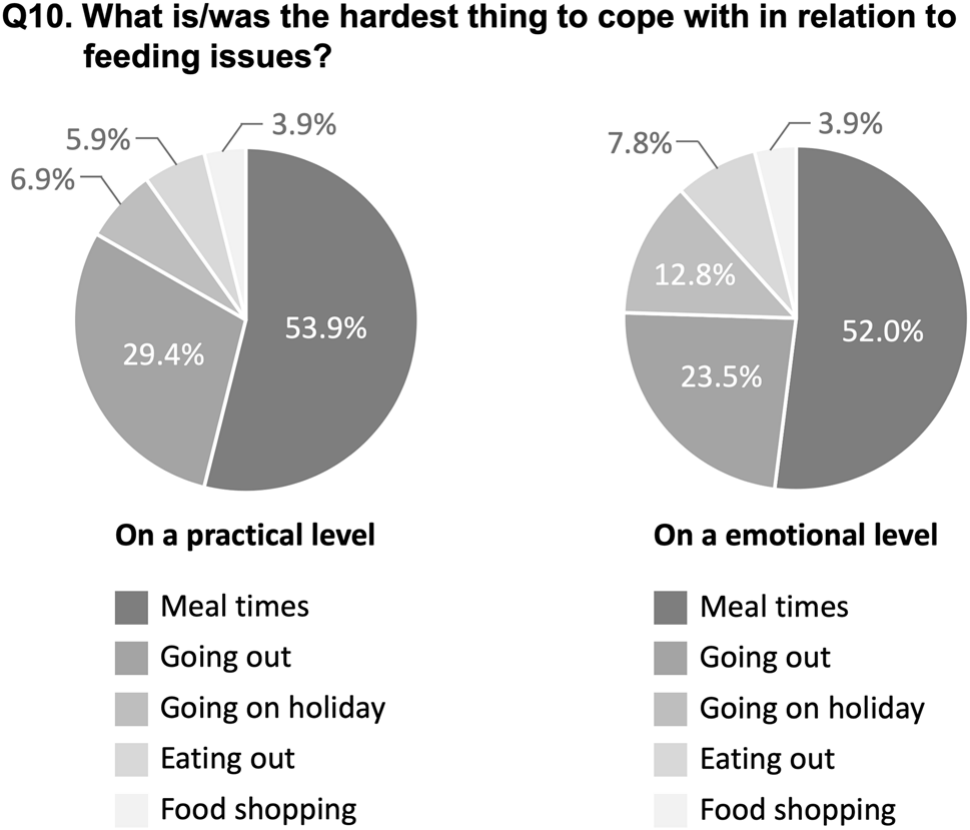
Pratical and emotional consequence to feeding issues

Overall, 116 (76.8%) responders reported suffering from any type of feeding issue (Fig. 1; Q3, Q6). Among those patients, about half of them (54/106, 50.9%) have been diagnosed with any feeding disability by medical team (Fig. 1; Q7). Quite worryingly, three fourths of the responders (78/106, 73.6%) did not agreed with the statement that feeding problems improved with time (Fig. 1; Q8). The commonest symptoms were gastroesophageal reflux (GER), low body weight, and swallowing difficulty (Fig. 2; Q4). When asked how their children’s attitude to food was, 48 (45.3%) responders described they are picky eaters, 33 (31.1%) are disinterested in food, 30 (28.3%) are aversive to textures of food, and 25 (24.6%) have aversion to food itself (Fig. 2; Q5). Feeding problems can be so severe that 28 (26.4%) patients have been supported at some stage by nasogastric tube, 25 (23.6%) needed a gastrostomy, and 2 (1.9%) have had received intravenous total parenteral nutrition (TPN) (Fig. 2; Q9). There was a proportion of activities which were hard to cope with, such as meal time, going holiday, and in general going out (Fig. 3; Q10).

### Daily life and support for feeding problems

**Fig. 4:**
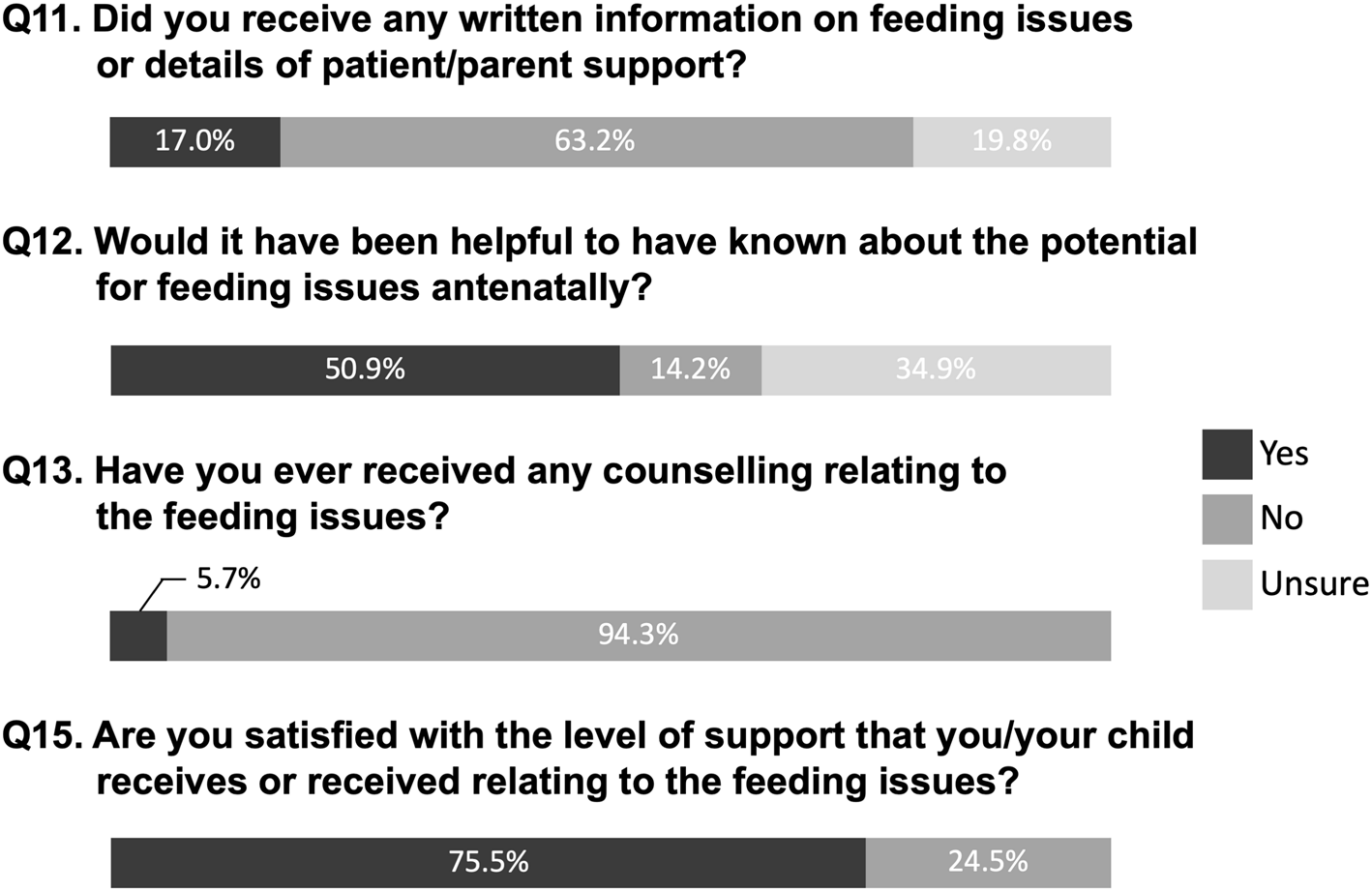
Support and information received

**Fig. 5:**
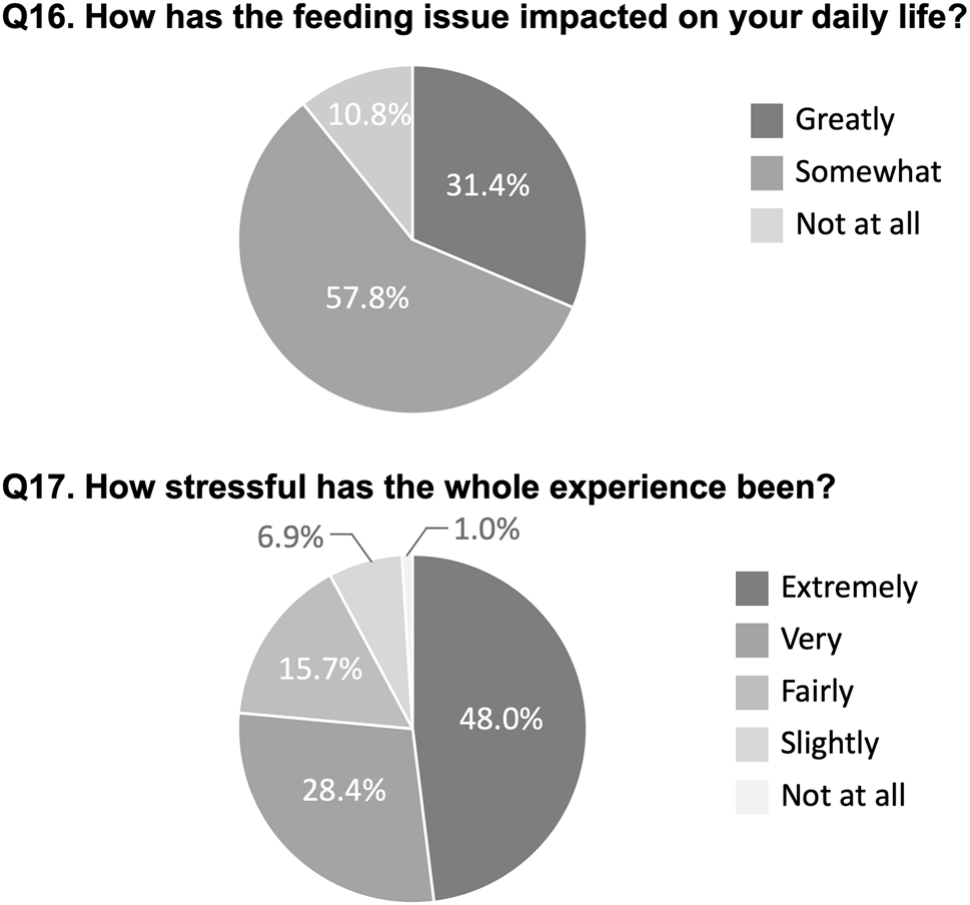
Impact on daily life

Despite feeding being an important issue, only a few families have received adequate support. Half of the patients (53/106, 50.0%) have visited a pediatric dietetics clinic, 43 (40.6%) have received speech and language therapy (SALT), 32 (30.2%) have attended a dedicated CDH clinic, and 10 (9.4%) have seen a specialist in a feeding clinic (Fig. 2; Q14). Only 18 (17.0%) responders have had any written information on feeding issues and have been offered any patient/parent support (Fig. 4; Q11). Only 6 (5.7%) patients received specialist counselling particularly to their feeding issue, and 5 (83.3%) of them described it as helpful (Fig. 4; Q13). Majority of responders (80/106, 75.5%) were satisfied with the level of support they were receiving (Fig. 4; Q15). Most of the responders (91/102, 89.2%) reported feeding problems impacted on their daily life, and 31.8% of them described it as “great impact” (Fig. 5; Q16). The majority (101/102, 99.0%) answered that the whole experience associated with the disease has been somewhat stressful and 78 (76.4%) responders described it has been very or extremely stressful (Fig. 5; Q17).

## Discussion

The advance of neonatal intensive care, including the gentle ventilation strategy with high frequently oscillation ventilator (HFOV) and the advent of ECMO, has improved the survival rate of patients with CDH, especially those who are in severe conditions [4–6]. At the same time, increasing number of children who have survived arduous neonatal period is giving rise to new issues related to long-time morbidity and transition of follow-up care [7–9]. There are evidences of high morbidity rate (60%) of CDH survivors and it was reported that they frequently experience growth delay (80%), gastric acid reflux (60%), recurrent pneumonia and chronic cough (10%), scoliosis (30%), and pectus deformity (20%) [3, 10–12]. Additionally, there are also neurological issues including developmental delay, which is potentially result from exposure to hypoxia and muscle relaxants in the early neonatal period [11, 13]. Hearing loss is another relevant neurological morbidity and reported to be observed in 20% of CDH survivors [13, 14]. A correlation between long-time sequelae and severity of CDH is generally acknowledged, but even patients who are successfully treated without ECMO nor patch repair are at risk of abovementioned symptoms [15–17]. Therefore, it is not straightforward to predict the long-time sequalae at the time of surgery and it is desirable to diligently follow up each patient in a comprehensive way. This is particularly relevant because of their impact on QOL of CDH patients and their families. For the first time, our group looked into those effects on the daily life CDH survivors. Thanks to the CDH UK, we could collect unique information which helped understanding patients’ perspective. The most important message is that, although it is usually kept within pre-clinical level, most of the patients and families are feeling stress in their daily activity. Feeding problems such as dysphagia and food intolerance seemingly have great impact on their QOL, by forcing the families to spend much time for feeding their children, which can put stress on their relationship.

The most common feeding issue is GER, being reported by over 90% of responders. Previous reports have described the incidence of GER in CDH survivors as 30–60% with variability depending on diagnostic criteria [18–20]. We assume the higher rate in our result is representing the recognition of the patients, reporting reflux as any vomit or regurgitation. Recently, multichannel intraluminal impedance (MII), which objectively detects the non-acidic reflux, has been introduced and revealed the high prevalence of non-acidic reflux at 80% [21]. Moreover, impaired motility of esophagus after CDH repair has been identified to induce prolonged clearance time, which subjects lower esophagus to risk of chronic esophagitis due to excess acid exposure. Even though patients have no typical symptom, GER possibly exist and silent reflux is a risk factor of later complications, such as Barrett’s esophagitis and consequent adenocarcinoma [20, 22, 23]. These facts indicate that GER is common, but non-negligible condition, recommending clinicians to carefully detect a sign of reflux and apply antipeptic therapy at proper timing. Fortunately, it is reported that the incidence of GER decreases after 1 year of age [18, 21].

Growth retardation is also problematic and experienced by 60% of the CDH survivors. Although increased catabolic stress and restricted water intake based on pulmonary impairment are likely to have some influence, the major factor of delayed growth is probably the lack in nutrition related to dysphagia, GER, and oral aversion [12]. The important thing is to routinely assess patient’s nutritional condition and find any sign of nutritional deficiency. The etiology of oral aversion is unspecified, but there is the speculation that long history of intubation and delayed oral feeding affect the mouth sensitivity [16]. In addition, delay in initiation of oral feeding related to dependence on tube feeding can influence developmental delay [11]. Further negative point of dysphagia is its impact on the QOL, indicated by the fact that more than half of the responders complained the meal time is the hardest thing to cope with. It is not hard to imagine how oral aversion confuses and frustrates parents. However, there is hopeful data that oral aversion tends to improve gradually coincided with the start of school or social activity [16]. In addition, early intervention of nutritional support and rehabilitation may have potential to moderate this issue.

As CDH survivors are vulnerable to various problems and remain complex beyond the neonatal period, the importance of multidisciplinary team (MDT) approach is increasing. MDT usually consists of different type of professionals including pediatric surgeon, pediatrician, pulmonologist, cardiologist, neurodevelopmentalist, dietitian, and so on, smoothing comprehensive assessment of patients in complicated condition. MDT clinics specifically designed for particular congenital disease, such as CDH, anorectal malformation, and abdominal wall defects have been gradually increasing. Moreover, the trial to consolidate post-discharge care of CDH in MDT clinic, so called CDH clinic, has been under way in some areas [10, 17]. Unfortunately, according to our survey, only one third of the patients have ever attended a CDH clinic, indicating constructing effective networks which facilitates easy access to MDT care will be a future subject. One of the concerning findings of this study is that very few patients received sufficient information on feeding problems and related supporting resource. This creates anxiety to CDH families who find it very difficult to cope with this long-term problem. Informing not only about survival, but also long-time outcome in advance helps the parents imagine the daily life after discharge of the baby and prepare themselves emotionally [24]. In our opinion, this should be discussed early with the family, even before birth.

This research has some limitations due to the patient-led nature of the survey. The subject of the study included only the registered members, thus the data doesn’t represent all cohort of CDH survivors, and incompleteness of questionnaire in a third of the responders potentially skews the data. Although the data are lacking detailed clinical information, such as age, weight, and operative procedure, direct responses assembled by volunteers are valuable, helping surgeons consider the transitional care for CDH survivor form the different aspect.

## Conclusion

This is the first patient-led survey focusing on feeding problems in the daily life of CDH survivors. CDH survivors frequently have various issues with feeding, which may not be adequately supported or discussed clinically. It is desirable to assist the patients to reliable resources of long-term support and MDT clinics may play an important role to accomplish it.

## References

[1] Harting MT, Lally KP (2014). The Congenital Diaphragmatic Hernia Study Group registry update. Semin Fetal Neonatal Med.

[2] McGivern MR, Best KE, Rankin J (2015). Epidemiology of congenital diaphragmatic hernia in Europe: a register-based study. Arch Dis Child Fetal Neonatal Ed.

[3] Ali K, Dassios T, Syed K (2019). Outcomes of infants with congenital diaphragmatic hernia by side of defect in the FETO era. Pediatr Surg Int.

[4] Ramakrishnan R, Salemi JL, Stuart AL (2018). Trends, correlates, and survival of infants with congenital diaphragmatic hernia and its subtypes. Birth Defects Res.

[5] Shanmugam H, Brunelli L, Botto LD (2017). Epidemiology and prognosis of congenital diaphragmatic hernia: a population-based cohort study in Utah. Birth Defects Res.

[6] Downard CD, Jaksic T, Garza JJ (2003). Analysis of an improved survival rate for congenital diaphragmatic hernia. J Pediatr Surg.

[7] West SD, Wilson JM (2005). Follow up of infants with congenital diaphragmatic hernia. Semin Perinatol.

[8] Chiu P, Hedrick HL (2008). Postnatal management and long-term outcome for survivors with congenital diaphragmatic hernia. Prenat Diagn.

[9] Poley MJ, Stolk EA, Tibboel D (2004). Short term and long term health related quality of life after congenital anorectal malformations and congenital diaphragmatic hernia. Arch Dis Child.

[10] Jancelewicz T, Chiang M, Oliveira C, Chiu PP (2013). Late surgical outcomes among congenital diaphragmatic hernia (CDH) patients: Why long-term follow-up with surgeons is recommended. J Pediatr Surg.

[11] Wynn J, Aspelund G, Zygmunt A (2013). Developmental outcomes of children with congenital diaphragmatic hernia: a multicenter prospective study. J Pediatr Surg.

[12] Safavi A, Synnes AR, O’Brien K (2012). Multi-institutional follow-up of patients with congenital diaphragmatic hernia reveals severe disability and variations in practice. J Pediatr Surg.

[13] Lund DP, Mitchell J, Kharasch V (1994). Congenital diaphragmatic hernia: the hidden morbidity. J Pediatr Surg.

[14] Wilson MG, Riley P, Hurteau AM (2013). Hearing loss in congenital diaphragmatic hernia (CDH) survivors: is it as prevalent as we think?. J Pediatr Surg.

[15] McGahren ED, Mallik K, Rodgers BM (1997). Neurological outcome is diminished in survivors of congenital diaphragmatic hernia requiring extracorporeal membrane oxygenation. J Pediatr Surg.

[16] Muratore CS, Utter S, Jaksic T (2001). Nutritional morbidity in survivors of congenital diaphragmatic hernia. J Pediatr Surg.

[17] Muratore CS, Kharasch V, Lund DP (2001). Pulmonary morbidity in 100 survivors of congenital diaphragmatic hernia monitored in a multidisciplinary clinic. J Pediatr Surg.

[18] Arcos-Machancoses JV, Ruiz Hernández C, Martin De Carpi J, Pinillos Pisón S (2018). A systematic review with meta-analysis of the prevalence of gastroesophageal reflux in congenital diaphragmatic hernia pediatric survivors. Dis Esophagus.

[19] Peetsold MG, Kneepkens CF, Heij HA (2010). Congenital diaphragmatic hernia: long-term risk of gastroesophageal reflux disease. J Pediatr Gastroenterol Nutr.

[20] Vanamo K, Rintala RJ, Lindahl H, Louhimo I (1996). Long-term gastrointestinal morbidity in patients with congenital diaphragmatic defects. J Pediatr Surg.

[21] Caruso AM, Di Pace MR, Catalano P (2013). Gastroesophageal reflux in patients treated for congenital diaphragmatic hernia: short- and long-term evaluation with multichannel intraluminal impedance. Pediatr Surg Int.

[22] Steven MJ, Fyfe AHB, Raine PAM, Watt I (2007). Esophageal adenocarcinoma: a long-term complication of congenital diaphragmatic hernia?. J Pediatr Surg.

[23] Fasching G, Huber A, Uray E (2000). Gastroesophageal reflux and diaphragmatic motility after repair of congenital diaphragmatic hernia. Eur J Pediatr Surg.

[24] Aite L, Trucchi A, Nahom A (2004). Antenatal diagnosis of diaphragmatic hernia: parents’ emotional and cognitive reactions. J Pediatr Surg.

